# A first faunistic study on the tribe Oniticellini Kolbe, 1905 (Coleoptera: Scarabaeidae) of Baikunthapur Tropical Forest of the Himalayan foothills, West Bengal, India

**DOI:** 10.3897/BDJ.8.e57444

**Published:** 2020-12-03

**Authors:** Subhankar Kumar Sarkar, Bhim Prasad Kharel

**Affiliations:** 1 Entomology Laboratory, Department of Zoology, University of Kalyani, Kalyani - 741235, West Bengal, India Entomology Laboratory, Department of Zoology, University of Kalyani Kalyani - 741235, West Bengal India

**Keywords:** distribution, diversity, dung beetles, oriental, taxonomy

## Abstract

**Background:**

Oniticellini Kolbe, 1905 is a paucispecific tribe of the scarab beetle subfamily Scarabaeinae. The tribe is composed of 256 described species worldwide, while from India, 26 species were recorded to date. Beetles belonging to this tribe are commonly known as paracoprid dung beetles and perform some remarkable ecological functions. Nevertheless, there is a dearth of knowledge on the occurrence of these beetles in the mega diverse tropical forests of the Himalayan foothills located in the north of the West Bengal state of India.

**New information:**

A first faunistic account of the tribe Oniticellini Kolbe, 1905 from Baikunthapur Forest, located at the Himalayan foothills of the West Bengal state of India is presented. A total of five species of the tribe distributed over two genera *Tiniocellus* and *Liatongus* were recorded during multiple surveys of the scarab fauna of the Forest. All taxa were recorded for the first time from the area, while *Tiniocellus spinipes* (Roth, 1851) is a new record for the West Bengal State of India. Additionally, a preliminary checklist of Indian species of the tribe is also provided.

## Introduction

The tribe Oniticellini Kolbe, 1905 shows a cosmopolitan distribution and is composed of approximately 26 genera and 252 species worldwide (Philips 2016). Currently, the tribe has four subtribes, namely Drepanocerina van Lansberge, 1875, Oniticellina Kolbe, 1905, Helictopleurina Janssens, 1946 and Eurysternina Volcano, Martinez & Pereira, 1960 (Branco 2010, Philips 2016). The subtribe Drepanocerina is composed of 11 genera and 46 species, the subtribe Oniticellina of 10 genera and 91 species, the subtribe Helictopleurina of two genera and 62 species and the subtribe Eurysternina of one genus and 53 species worldwide (Schoolmeesters 2020).

Beetles of this tribe perform some indispensible ecological functions to the forest ecosystem. According to their nesting strategies and ecological role, they are called tunnellers and belong to the paracoprid functional guild of coprophagous beetles (Halffter and Edmonds 1982). These beetles, while feeding and nesting, enhance nitrogen volatilisation and mineralisation rates by altering the microorganism fauna present in dung pats and brood balls (Yokoyama et al. 1991). Adults and larvae of these beetles also control the abundance of dung breeding detrivorous flies and dung dispersed nematodes and protozoa (Nichols et al. 2008). These ecological functions have immense implications for livestock, wildlife and human health (Miller 1954, Byford et al. 1992). Considering all these functions, the paracoprid beetles form an extremely important component of tropical forest ecosystems.

From the Oriental Region, the first ever comprehensive work on the taxonomy of Scarabaeidae was that of Arrow (1910), Arrow (1917), Arrow (1931). Later, monographic works from the Oriental and Palaearctic Regions were presented by Balthasar (1963a), Balthasar (1963b), Balthasar (1963c), Oppenheimer (1977), Micsik (1982), Lumaret and Lobo (1996) and Löbl and Löbl (2016). From India, data on the occurrence of these beetles are chiefly based on the publications of the Zoological Survey of India and the works carried out regionally from different parts of India. Some of the noteworthy works are of Biswas and Chatterjee (1985), Chatterjee and Biswas (2000), Chandra (2005), Sewak (2006), Sewak (2009), Chandra and Ahirwar (2007), Karimbumkara and Priyadarsanan (2013), Sathiandran et al. (2015) and Gajendra and Prasad (2016). On the contrary, from the West Bengal State of India major contributions are of Sarkar et al. (2010) , Sarkar et al. (2014) , Sarkar et al. (2015) and Kharel et al. (2020). However, a concise taxonomic account of these beetles from India is still lacking.

The Baikunthapur Forest, although exhibiting the tropical forests of the Himalayan foothills and being a mega biodiversity zone, has never been assessed for its scarab fauna. The Forest is located at the south of the outer foothills of the Himalayas in the alluvial flood plains of the West Bengal State of India. The Forest stands as an excellent example of the tropical forest ecosystem of the Himalayan foothills and harbours an array of unique flora and fauna.

It is with this background that a project has been taken up to explore the scarab fauna of the Forest. Multiple surveys were carried out in different parts of the Forest, based on vegetation pattern and distribution of mammals. The surveys resulted in the recognition of approximately 78 scarab species, of which five belong to the tribe Oniticellini Kolbe, 1905 and are presented here in this paper. The five species belong to two genera *Liatongus* Reitter, 1893 and *Tiniocellus* Péringuey, 1901. *Liatongus* Reitter, 1893 is composed of 62 species worldwide, whereas *Tiniocellus* Péringuey, 1901 is a species-poor genus and has only seven species throughout the world (Schoolmeesters 2020). From India, the genus *Liatongus* is known by 10 species and *Tiniocellus* by two species.

Here, we compile a preliminary checklist of the Oniticellini species recorded from India to date (Table 1). The list is based on the distributional data published in past literature, including the publications of the Zoological Survey of India. Until now, 24 species were recorded from India, of which *Oniticellus cinctus* seems to be the most abundant species of the tribe across the country.

## Materials and methods

### Study site

**Figure 1.**
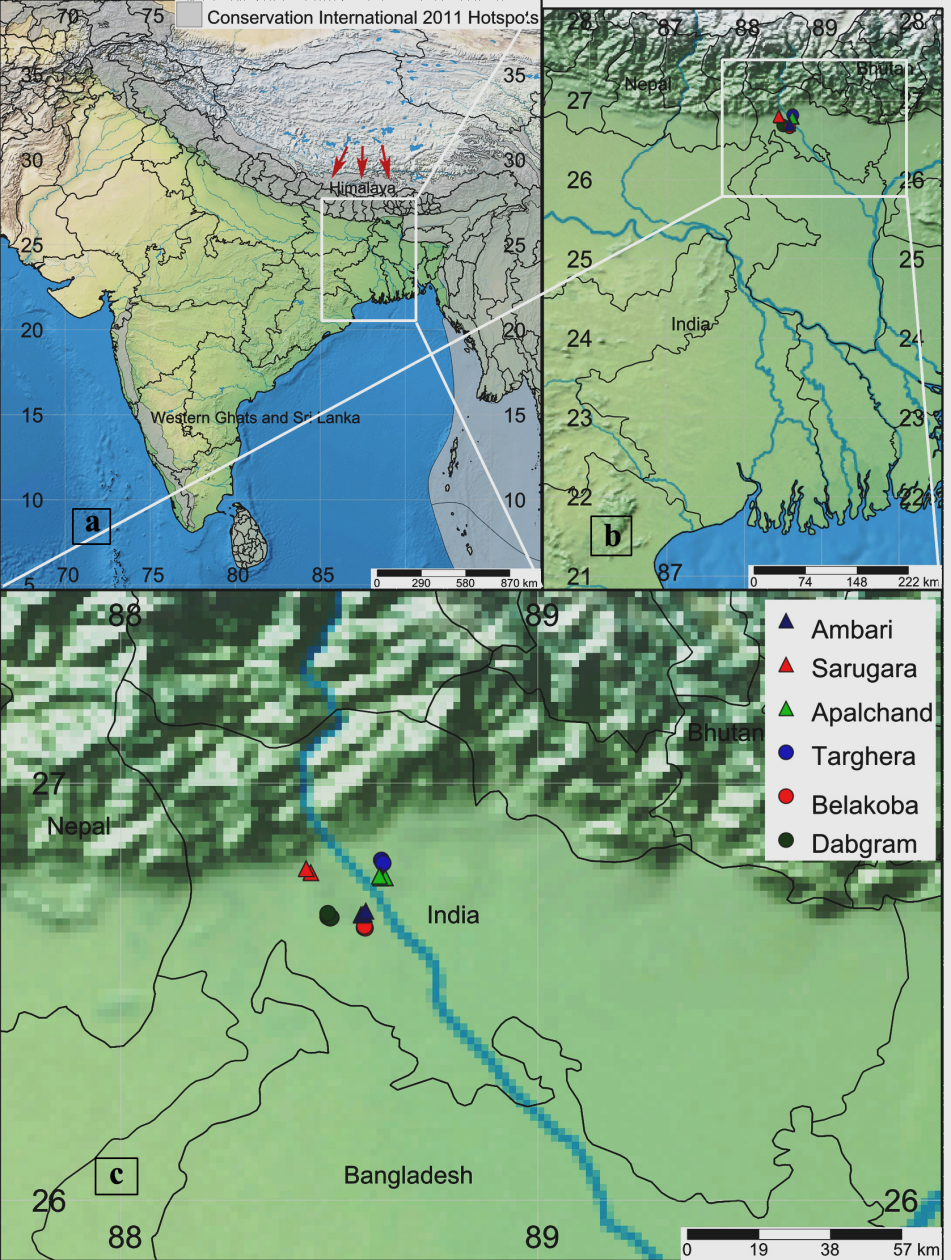
Map showing sampling localities in Baikunthapur Tropical Forest, West Bengal, India. **a.** Map of India showing West Bengal State (arrows indicating the Eastern Himalaya and the Biodiversity hotspot according to Conservation International 2011); **b.** West Bengal State showing the location of study area; **c.** Collection points in the study area.

The Baikunthapur Forest is located in the northern part of the West Bengal State of India (Fig. 1). It is a Terai forested area, situated at the Himalayan foothills in the Dooars Region of theState. The forested area forms an extremely important ecological zone and provides habitat for many wild animals, such as Elephant, Royal Bengal Tiger, One horned Rhinoceros, Barking Deer, Hog Deer, Bison, Chital, Wild Boar etc. The Forest, spread over an area of 257.13 km^2^, lies between 26°30' and 27°00' north latitude and 88°20' and 88°40' east longitude and has an annual rainfall of 382 cm. The altitude of the forested area ranges from 100 m to 165 m above mean sea level. The Mahananda River flows to its west, while the River Teesta to its east. The entire Forest is subdivided into six different forest ranges namely Ambari, Sarugara, Apalchand, Targhera, Belacoba, and Dabgram. The vegetation pattern of the Forest is characterised as riverine grassland and moist deciduous forest. The grassland comprises mainly of tall grasses, namely Kasia (*Saccharum spontaneum*), Dhadda (*Saccharum arundinaceum*), Nal (*Arundo donax*), Khagra (*Phragmites karka*) etc. Locally, these grasses are known as ‘elephant grass’ and can grow up to the height of 6 m. Some of the dominant tree species of the Forest are Sal (Shorea robusta), Kadam (*Anthocephalus cadamba*), Siris (*Albizzia procera*), Simul (*Bombax ceiba*), Khair (*Acacia catechu*), Chalta (*Dellenia indica*) etc.

### Specimen collection and identification

Several faunistic surveys were carried out at all of the six Forest ranges of the study area during March 2018–February 2020. Insect specimens were collected in every month from each range during the period of surveys. Random sampling, hand picking from dung pats and pitfall traps were utilised for the collection of beetles. The pitfall traps were made of plastic containers (210 mm in diameter and 150 mm in depth) and buried into the soil up to their rims. The traps contained water-formalin-liquid soap mixture, a wire grid above the mixture and about one litre of animal dung over the wire grid. The traps were placed in four replicates at all of the six ranges during our survey. Beetle specimens were collected from the traps after every 5–6 days of exposure in the field. After collection, the specimens were placed in a jar containing chloroform-dipped cotton and then transferred to 70% alcohol in glass vials. The male genitalia (aedeagus) was dissected out and cleaned in 10% potassium hydroxide (KOH) solution and the remaining muscles and fats were removed further in glycerine. Species identification was done following the keys of Arrow (1931) and Branco (2010). The photographs of adult habitus and genitalia were taken with the camera (MAGCAM DC 5) attached to the Stereozoom Trinocular Microscope (OLYMPUS SZX7). For measurements, the apex of clypeus to the tip of elytra and maximum width of the elytral base were taken for length and breadth, respectively. The map of the study area showing collection points was created online at simplemappr.com and compiled in Adobe Photoshop CS3.

### Material deposition

The collected beetle specimens were deposited in the Entomology Laboratory of the Department of Zoology, University of Kalyani, West Bengal, India (ZE-KU).

## Checklists

### Systematic account of the species recorded from Baikunthapur Tropical Forest

#### Scarabaeidae

Latreille, 1802

3C84D05B-238A-5C41-852B-6CD2AA98A3E1

#### Scarabaeinae

Latreille, 1802

619AEEED-5E97-5B29-8336-B77E93D36AF6

#### Oniticellini

Kolbe, 1905

2FFBCF02-5035-5E49-BEBD-438A5F81E788

#### Oniticellina

Kolbe, 1905

B31B21EC-456A-5047-AC2F-1FB234A292DA

#### Liatongus

Reitter, 1893

3FD421C8-04DF-52BC-8213-7DDF014CE21B

#### Liatongus affinis

(Arrow, 1908)

C8BCD6DC-C9AF-57C3-BA9E-0F6853C0D8CC

##### Materials

- **Type status:** Other material. **Occurrence:** recordedBy: Subhankar Kumar Sarkar; sex: 1 male; **Location:** country: India; countryCode: IND; stateProvince: West Bengal; municipality: Jalpaiguri; locality: Ambari range of Baikunthapur Forest; verbatimCoordinates: 26°41'3.21"N, 88°34'46.86"E; **Event:** samplingProtocol: Pitfall trap; eventDate: 19-Aug-18; habitat: Mammalian dung; **Record Level:** collectionCode: ZE-KU_BKF01; basisOfRecord: Preserved Specimen
- **Type status:** Other material. **Occurrence:** recordedBy: Subhankar Kumar Sarkar and Bhim Prasad Kharel; sex: 2 males; **Location:** country: India; countryCode: IND; stateProvince: West Bengal; municipality: Jalpaiguri; locality: Apalchand range of Baikunthapur Forest; verbatimCoordinates: 26°46'41.98"N, 88°37'26.35"E; **Event:** samplingProtocol: Hand picking from dung pats; eventDate: 03-Mar-19; habitat: Mammalian dung; **Record Level:** collectionCode: ZE-KU_BKF02, ZE-KU_BKF03; basisOfRecord: Preserved Specimen
- **Type status:** Other material. **Occurrence:** recordedBy: Subhankar Kumar Sarkar and Bhim Prasad Kharel; sex: 2 males; **Location:** country: India; countryCode: IND; stateProvince: West Bengal; municipality: Jalpaiguri; locality: Dabgram range of Baikunthapur Forest; verbatimCoordinates: 26°40'46.84"N, 88°29'47.24"E; **Event:** samplingProtocol: Hand picking from dung pats; eventDate: 05-Jan-20; habitat: Mammalian dung; **Record Level:** collectionCode: ZE-KU_BKF04, ZE-KU_BKF05; basisOfRecord: Preserved Specimen

##### Ecological interactions

###### Feeds on

Mammalian dung

##### Distribution

CHINA; INDIA: Assam, Karnataka, Manipur, Tripura, West Bengal; MYANMAR; THAILAND (Chatterjee and Biswas 2000, Karimbumkara and Priyadarsanan 2013, Schoolmeesters 2020).

##### Diagnosis

This species can be distinguished from other *Liatongus* species by the following combination of characters (Fig. 2a): Length 7 mm, breadth 4 mm; upper surface dark and moderately shining; head bears a broad transverse carina on the vertex with its angles forming a pair of sharp pointed divergent processes; clypeus bilobed at front and nearly straight at sides, moderately punctured and bears a curved carina posteriorly; pronotum dark brown on the disc, laterally testaceous, strongly and closely punctured, with a mid-longitudinal groove near the base and a slight vertical declivity in the middle anteriorly; elytra deeply striate, irregularly punctured, intervals slightly convex; metasternal shield finely punctured, mid-longitudinally grooved; pygidium coarsely punctured, surface coriaceous. Genitalia: Phallobase elongate, broad at base, heavily sclerotised (Fig. 3a); parameres broad, ventrally curved with the apex extended into a hook-like process (Fig. 3b).

#### Liatongus mergacerus

(Hope, 1831)

68911C1C-7FF4-5366-839C-7CC4F15F2F97

##### Materials

- **Type status:** Other material. **Occurrence:** recordedBy: Bhim Prasad Kharel; sex: 1 male; **Location:** country: India; countryCode: IND; stateProvince: West Bengal; municipality: Jalpaiguri; locality: Apalchand range of Baikunthapur Forest; verbatimCoordinates: 26°46'22.01"N, 88°37'22.38"E; **Event:** samplingProtocol: Random sampling; eventDate: 26-Oct-18; habitat: Mammalian dung; **Record Level:** collectionCode: ZE-KU_BKF06; basisOfRecord: Preserved Specimen
- **Type status:** Other material. **Occurrence:** recordedBy: Subhankar Kumar Sarkar; sex: 1 male; **Location:** country: India; countryCode: IND; stateProvince: West Bengal; municipality: Jalpaiguri; locality: Targhera range of Baikunthapur Forest; verbatimCoordinates: 26°48'56.20"N, 88°37'30.40"E; **Event:** samplingProtocol: Pitfall trap; eventDate: 08-Jun-18; habitat: Mammalian dung; **Record Level:** collectionCode: ZE-KU_BKF07; basisOfRecord: Preserved Specimen
- **Type status:** Other material. **Occurrence:** recordedBy: Subhankar Kumar Sarkar and Bhim Prasad Kharel; sex: 3 males; **Location:** country: India; countryCode: IND; stateProvince: West Bengal; municipality: Jalpaiguri; locality: Ambari range of Baikunthapur Forest; verbatimCoordinates: 26°41'1.15"N, 88°34'33.10"E; **Event:** samplingProtocol: Hand picking from dung pats; eventDate: 22-Jun-18; habitat: Mammalian dung; **Record Level:** collectionCode: ZE-KU_BKF08, ZE-KU_BKF09, ZE-KU_BKF10; basisOfRecord: Preserved Specimen

##### Ecological interactions

###### Feeds on

Mammalian dung

##### Distribution

BHUTAN; INDIA: Arunachal Pradesh, Sikkim, Uttarakhand, Uttar Pradesh, West Bengal; NEPAL; SUDAN (Biswas and Chatterjee 1985, Löbl and Smetana 2006, Sewak 2006, Schoolmeesters 2020).

##### Diagnosis

This species can be distinguished from other *Liatongus* species by the following combination of characters (Fig. 2b): Length 9 mm, breadth 5.5 mm; upper surface dark brown and opaque; head finely punctured with the sides round, forehead bears a strong, basally flat and backwardly curved, erect horn, with its tip diverged into two slender and sharply pointed processes; clypeus finely punctured and separated from the forehead by a feebly curved carina; pronotum very deeply and broadly excavated in its anterior part with the cavity smooth, shining and posteriorly elevated into a strong, straight and compressed, blade-like horn, with its tip rounded; elytra deeply striate and strongly punctured, intervals slightly convex; metasternal shield very smooth and bears a mid-longitudinal groove; pygidium minutely punctured. Genitalia: Phallobase elongate, broad at base and heavily sclerotised (Fig. 3c); parameres ventrally curved, broad and round at base, laterally extended into a hook-like process, apically sinuate with the tips round and blunt (Fig. 3d).

#### Liatongus rhinoceros

Arrow, 1931

F20C94B2-83DB-5413-B549-65C2647CC2FC

##### Materials

- **Type status:** Other material. **Occurrence:** recordedBy: Subhankar Kumar Sarkar and Bhim Prasad Kharel; sex: 2 females; **Location:** country: India; countryCode: IND; stateProvince: West Bengal; municipality: Jalpaiguri; locality: Belacoba range of Baikunthapur Forest; verbatimCoordinates: 26°39'13.46"N, 88°35'1.41"E; **Event:** samplingProtocol: Hand picking from dung pats; eventDate: 28-Feb-19; habitat: Mammalian dung; **Record Level:** collectionCode: ZE-KU_BKF11, ZE-KU_BKF12; basisOfRecord: Preserved Specimen
- **Type status:** Other material. **Occurrence:** recordedBy: Subhankar Kumar Sarkar; sex: 1 female; **Location:** country: India; countryCode: IND; stateProvince: West Bengal; municipality: Jalpaiguri; locality: Sarugara range of Baikunthapur Forest; verbatimCoordinates: 26°47'7.1"N, 88°27'20.2"E; **Event:** samplingProtocol: Random sampling; eventDate: 29-Mar-19; habitat: Mammalian dung; **Record Level:** collectionCode: ZE-KU_BKF13; basisOfRecord: Preserved Specimen
- **Type status:** Other material. **Occurrence:** recordedBy: Subhankar Kumar Sarkar; sex: 1 female; **Location:** country: India; countryCode: IND; stateProvince: West Bengal; municipality: Jalpaiguri; locality: Dabgram range of Baikunthapur Forest; verbatimCoordinates: 26°40'40.33"N, 88°30'16.92"E; **Event:** samplingProtocol: Pitfall trap; eventDate: 16-Feb-20; habitat: Mammalian dung; **Record Level:** collectionCode: ZE-KU_BKF14; basisOfRecord: Preserved Specimen

##### Ecological interactions

###### Feeds on

Mammalian dung

##### Distribution

INDIA: Himachal Pradesh, Sikkim, West Bengal; NEPAL; SUDAN (Chandra 2005, Löbl and Smetana 2006, Schoolmeesters 2020).

##### Diagnosis

This species can be distinguished from other *Liatongus* species by the following combination of characters (Fig. 2c): Length 7 mm, breadth 3.5 mm; upper surface dark; head broad, angulate at sides, finely punctured; pronotum coarsely and densely punctured, with a mid-longitudinal groove at the base and a curved declivity just behind the front margin, the edge of the declivity raised and forming a pair of strong tubercles on each side; elytra shallowly striate, closely punctured, with the intervals flat; metasternal shield smooth in the middle and bears a large depression upon the posterior part; pygidium densely setose and closely punctured.

#### Tiniocellus

Péringuey, 1901

94155496-0705-58C5-87AC-D3311058F9F0

#### Tiniocellus imbellis

(Bates, 1891)

AF878B4E-9B27-5BBC-8F31-655045BD6016

##### Materials

- **Type status:** Other material. **Occurrence:** recordedBy: Subhankar Kumar Sarkar and Bhim Prasad Kharel; sex: 3 females; **Location:** country: India; countryCode: IND; stateProvince: West Bengal; municipality: Jalpaiguri; locality: Sarugara range of Baikunthapur Forest; verbatimCoordinates: 26°47'41.2"N, 88°26'40.3"E; **Event:** samplingProtocol: Hand picking from dung pats; eventDate: 24-Mar-18; habitat: Mammalian dung; **Record Level:** collectionCode: ZE-KU_BKF15, ZE-KU_BKF16, ZE-KU_BKF17; basisOfRecord: Preserved Specimen
- **Type status:** Other material. **Occurrence:** recordedBy: Subhankar Kumar Sarkar; sex: 1 female; **Location:** country: India; countryCode: IND; stateProvince: West Bengal; municipality: Jalpaiguri; locality: Apalchand range of Baikunthapur Forest; verbatimCoordinates: 26°46'23.71"N, 88°38'3.46"E; **Event:** samplingProtocol: Pitfall trap; eventDate: 26-Oct-18; habitat: Mammalian dung; **Record Level:** collectionCode: ZE-KU_BKF18; basisOfRecord: Preserved Specimen
- **Type status:** Other material. **Occurrence:** recordedBy: Subhankar Kumar Sarkar and Bhim Prasad Kharel; sex: 3 females; **Location:** country: India; countryCode: IND; stateProvince: West Bengal; municipality: Jalpaiguri; locality: Belakoba range of Baikunthapur Forest; verbatimCoordinates: 26°39'9.56"N, 88°35'11.36"E; **Event:** samplingProtocol: Random sampling; eventDate: 04-Nov-18; habitat: Mammalian dung; **Record Level:** collectionCode: ZE-KU_BKF19, ZE-KU_BKF20, ZE-KU_BKF21; basisOfRecord: Preserved Specimen
- **Type status:** Other material. **Occurrence:** recordedBy: Subhankar Kumar Sarkar and Bhim Prasad Kharel; sex: 2 females; **Location:** country: India; countryCode: IND; stateProvince: West Bengal; municipality: Jalpaiguri; locality: Dabgram range of Baikunthapur Forest; verbatimCoordinates: 26°40'35.86"N, 88°30'7.66"E; **Event:** samplingProtocol: Pitfall trap; eventDate: 13-Feb-19; habitat: Mammalian dung; **Record Level:** collectionCode: ZE-KU_BKF22, ZE-KU_BKF23; basisOfRecord: Preserved Specimen
- **Type status:** Other material. **Occurrence:** recordedBy: Subhankar Kumar Sarkar; sex: 1 female; **Location:** country: India; countryCode: IND; stateProvince: West Bengal; municipality: Jalpaiguri; locality: Targhera range of Baikunthapur Forest; verbatimCoordinates: 26°48'30.22"N, 88°37'44.25"E; **Event:** samplingProtocol: Random sampling; eventDate: 08-Apr-19; habitat: Mammalian dung; **Record Level:** collectionCode: ZE-KU_BKF24; basisOfRecord: Preserved Specimen
- **Type status:** Other material. **Occurrence:** recordedBy: Subhankar Kumar Sarkar; sex: 4 female; **Location:** country: India; countryCode: IND; stateProvince: West Bengal; municipality: Jalpaiguri; locality: Ambari range of Baikunthapur Forest; verbatimCoordinates: 26°41'10.67"N, 88°35'1.33"E; **Event:** samplingProtocol: Hand picking from dung pats; eventDate: 22-Jun-19; habitat: Mammalian dung; **Record Level:** collectionCode: ZE-KU_BKF25, ZE-KU_BKF26, ZE-KU_BKF27, ZE-KU_BKF28; basisOfRecord: Preserved Specimen

##### Ecological interactions

###### Feeds on

Mammalian dung

##### Distribution

INDIA: Bihar, Himachal Pradesh, Karnataka, Kerala, Madhya Pradesh, Maharashtra, Punjab, Sikkim, Tamil Nadu, Uttar Pradesh, West Bengal; NEPAL; PAKISTAN (Branco 2010, Schoolmeesters 2020).

##### Diagnosis

This species can be distinguished from other *Tiniocellus* species by the following combination of characters (Fig. 2d): Length 6.5 mm, breadth 3 mm; upper surface dark brown; head short and broad, both coarsely and finely punctured with the large punctures setiferous, with the genae protruding from sides of clypeus at clypeo-genal junction and the vertex bears two sinuate carina basally; clypeus rugosely punctured, anteriorly reflexed and feebly excised in the middle; pronotum coarsely and closely punctured, disc without testaceous patches and bears a shallow median basal furrow occupying approximately half of its length; elytra not completely covering the abdomen and met-episterna at the sides, shallowly striate, interstriae flat, wide and fringed before the hind margin, 1^st^ interstria with four and 5^th^ with three long erect setae, 3^rd^ and 7^th^ without long erect setae other than the tuft on apical declivity; metasternal shield smooth, with a spoon shaped concavity at the end of impunctate and shining mid-line; pygidium wider at base than the 1^st^ to 5^th^ elytral interstriae of the two elytra taken together; hind femur devoid of setae on postero-inferior edge.

#### Tiniocellus spinipes

(Roth, 1851)

B1CBE606-08C3-52BE-8D6F-AED83710D6FA

##### Materials

- **Type status:** Other material. **Occurrence:** recordedBy: Bhim Prasad Kharel; sex: 1 female; **Location:** country: India; countryCode: IND; stateProvince: West Bengal; municipality: Jalpaiguri; locality: Ambari range of Baikunthapur Forest; verbatimCoordinates: 26°41'35.99"N, 88°35'15.02"E; **Event:** samplingProtocol: Hand picking from dung pats; eventDate: 27-Jul-18; habitat: Mammalian dung; **Record Level:** collectionCode: ZE-KU_BKF29; basisOfRecord: Preserved Specimen
- **Type status:** Other material. **Occurrence:** recordedBy: Bhim Prasad Kharel; sex: 1 female; **Location:** country: India; countryCode: IND; stateProvince: West Bengal; municipality: Jalpaiguri; locality: Apalchand range of Baikunthapur Forest; verbatimCoordinates: 26°46'37.34"N, 88°37'12.63"E; **Event:** samplingProtocol: Pitfall trap; eventDate: 27-Sep-18; habitat: Mammalian dung; **Record Level:** collectionCode: ZE-KU_BKF30; basisOfRecord: Preserved Specimen
- **Type status:** Other material. **Occurrence:** recordedBy: Subhankar Kumar Sarkar; sex: 1 female; **Location:** country: India; countryCode: IND; stateProvince: West Bengal; municipality: Jalpaiguri; locality: Dabgram range of Baikunthapur Forest; verbatimCoordinates: 26°41'16.01"N, 88°29'47.62"E; **Event:** samplingProtocol: Pit fall trap; eventDate: 12-Jan-19; habitat: Mammalian dung; **Record Level:** collectionCode: ZE-KU_BKF31; basisOfRecord: Preserved Specimen
- **Type status:** Other material. **Occurrence:** recordedBy: Subhankar Kumar Sarkar; sex: 2 females; **Location:** country: India; countryCode: IND; stateProvince: West Bengal; municipality: Jalpaiguri; locality: Belakoba range of Baikunthapur Forest; verbatimCoordinates: 26°39'24.40"N, 88°35'4.01"E; **Event:** samplingProtocol: Random sampling; eventDate: 28-Dec-19; habitat: Mammalian dung; **Record Level:** collectionCode: ZE-KU_BKF32, ZE-KU_BKF33; basisOfRecord: Preserved Specimen

##### Ecological interactions

###### Feeds on

Mammalian dung

##### Distribution

ANGOLA; BOTSWANA; BURKINA FASO; ERITREA; ETHIOPIA; GHANA; INDIA: Chhattisgarah, Gujarat, Haryana, Himachal Pradesh, Karnataka, Kerala, Madhya Pradesh, Maharashtra, Uttarakhand, Uttar Pradesh; IVORY COAST; KENYA; MALAWI; MOZAMBIQUE; NAMIBIA; PAKISTAN; REPUBLIC DEMOCRATIC CONGO; REPUBLIC OF GUINEA; REPUBLIC SOUTH AFRICA; SENEGAL; SOMALIA; TANZANIA; UGANDA; ZAMBIA; ZIMBABWE (Péringuey 1901, Branco 2010, Thomas et al. 2011, Chandra and Gupta 2013, Karimbumkara and Priyadarsanan 2013, Löbl and Smetana 2006, Noureen et al. 2015, Gajendra and Prasad 2016, Philips 2016, Anto and Vinod 2017, Singh et al. 2017, Schoolmeesters 2020).

##### Diagnosis

This species can be distinguished from other *Tiniocellus* species by the following combination of characters (Fig. 2e): Length 6 mm, breadth 2.5 mm; upper surface dark; head short and broad, gently rounded at sides, with the vertex bearing two sinuate carina basally and the genae not protruding from sides of clypeus at clypeo-genal junction; clypeus anteriorly reflexed and deeply bilobed in the middle; pronotum coarsely and rugosely punctured, very dark on disc and testaceous at sides and bears a mid-longitudinal groove at the base; elytra shallowly striate and not completely covering the abdomen and metepisterna at the sides, interstriae flat, narrow and fringed before the hind margin, 1^st^ interstria with 4, 5^th^ with 2 and 7^th^ with 1 long erect setae, 3^rd^ without long erect setae, other than the tuft on apical declivity; metasternal shield smooth, with a very indistinct depression medially; pygidium narrower at base than the 1^st^ to 5^th^ elytral interstriae of the two elytra taken together; hind femur devoid of setae on postero-inferior edge.

## Discussion

Beetles of the subfamily Scarabaeinae, commonly known as coprophagous or dung beetles, are broadly classified on the basis of their feeding and nesting strategies into three categories, namely paracoprid (tunnellers), endocoprid (dwellers) and telecoprid (rollers) dung beetles (Halffter and Edmonds 1982). The paracoprids bury their brood balls in vertical chambers (tunnels) near the original deposition site, the endocoprids brood their young inside the dung mass itself and the telecoprids transport and roll dung balls horizontally to some distance before their burial into the soil (Halffter and Edmonds 1982). By consuming and burying dung, these beetles play a significant role in various ecological functions, such as nutrient recycling, seed dispersal, bioturbation, pollination and parasite suppression (Yokoyama et al. 1991, Mittal 1993, Nichols et al. 2008). Moreover, these beetles, by altering the micro-organism fauna in dung pats, brood balls and associated soils, also stimulate the aerobic conditions and reduce methane production (Yokoyama et al. 1991, Mittal 1993, Nichols et al. 2008). Dung beetles show their highest diversity in tropical forests and savannahs (Hanski and Cambefort 1991) and the ecological functions performed by them are considered extremely important for the maintenance of biodiversity in these mega biodiversity zones.

During our survey, we have recorded 78 scarab species, of which 32 are dung beetles and belong to the subfamily Scarabaeinae. Of these, five included within the tribe Oniticellini, are paracoprid dung beetles and are presented here in this paper. All the five species were recorded for the first time from the area, while *Tiniocellus spinipes* is a new record for the West Bengal State of India. Amongst all recorded species, *Tiniocellus imbellis* seems to be the dominant species of the Forest as it shows maximum abundance and was recorded from maximum ranges of the Forest and is widely distrtibuted throughout the study area, followed by *Tiniocellus spinipes*.

We have also compiled one preliminary checklist of Indian species of the tribe Oniticellini (Table 1). The checklist is based on the distribution data published in past literature, including the papers published from the Zoological Survey of India. Until now, 24 species are known to occur across India, of which *Oniticellus cinctus* shows the widest distribution throughout the country as it is reported from a maximum number of States (17) followed by *Tibiodrepanus setosus* (13). From the West Bengal State, 10 species were recorded until now, which is about one half of the total known species of India. Of the 24 species presented in the Indian checklist, four show distribution only in India which reflect 17% endemism.

## Supplementary Material

XML Treatment for Scarabaeidae

XML Treatment for Scarabaeinae

XML Treatment for Oniticellini

XML Treatment for Oniticellina

XML Treatment for Liatongus

XML Treatment for Liatongus affinis

XML Treatment for Liatongus mergacerus

XML Treatment for Liatongus rhinoceros

XML Treatment for Tiniocellus

XML Treatment for Tiniocellus imbellis

XML Treatment for Tiniocellus spinipes

## Figures and Tables

**Figure 2a.**
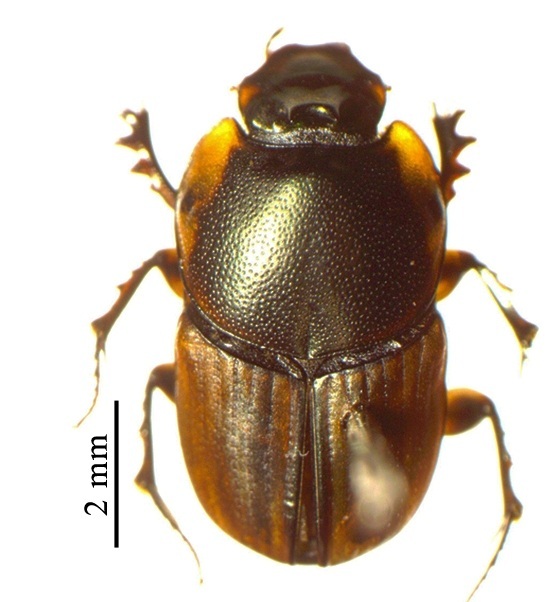


**Figure 2b.**
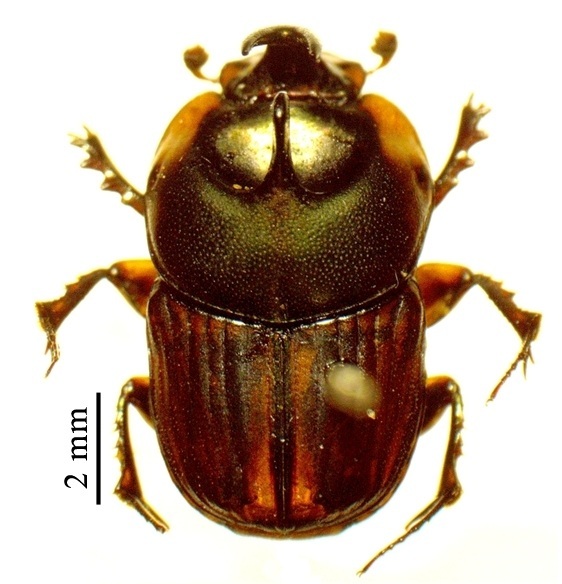


**Figure 2c.**
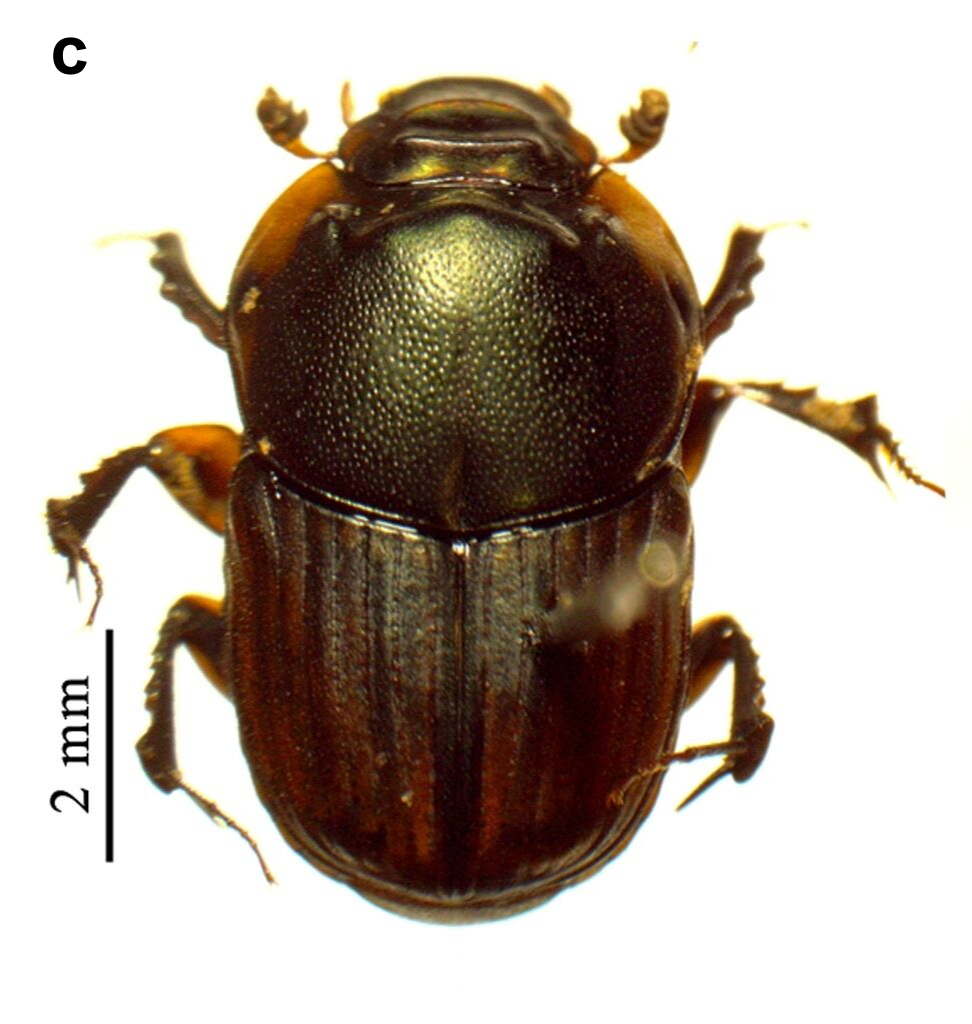


**Figure 2d.**
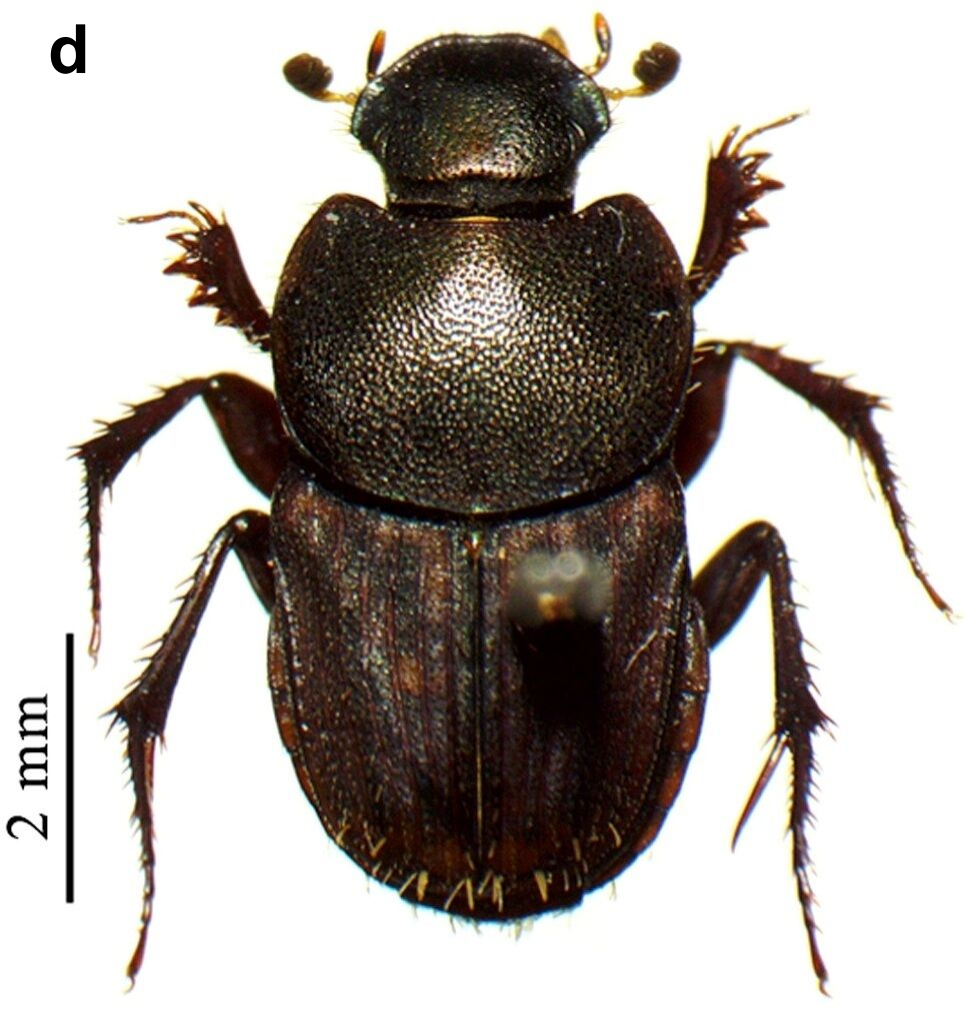


**Figure 2e.**
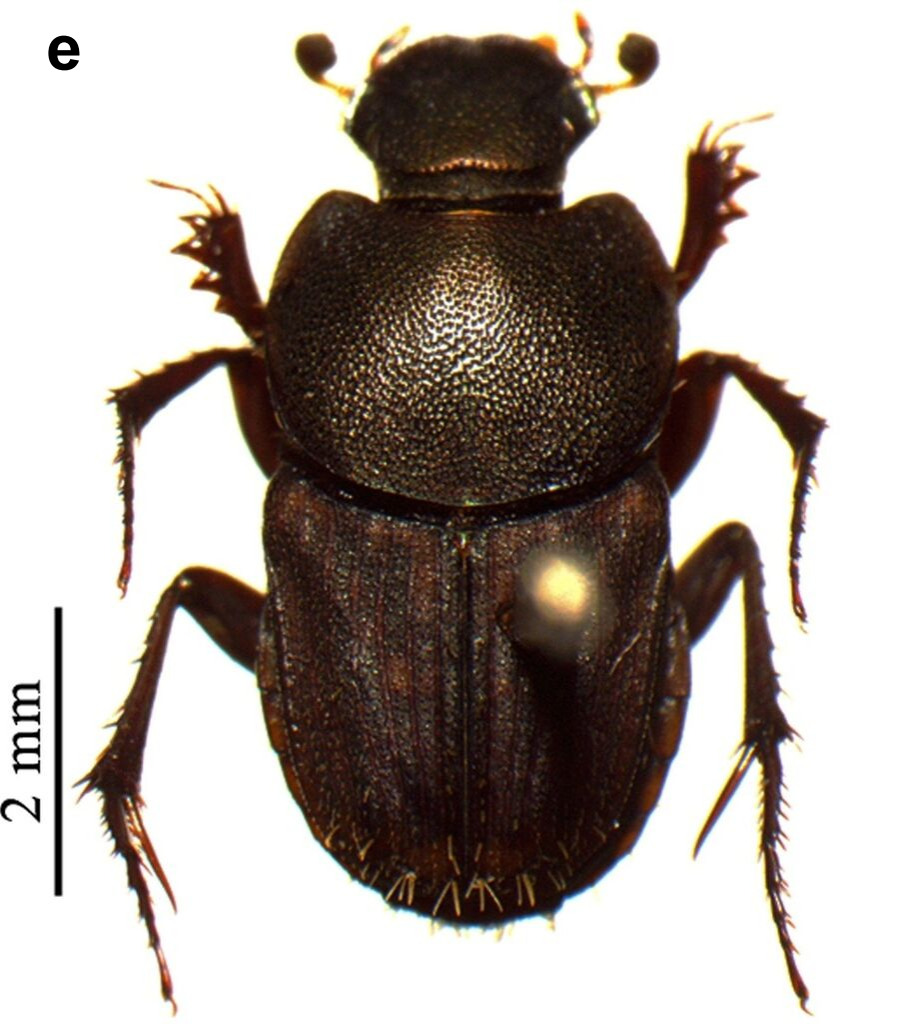


**Figure 3a.**
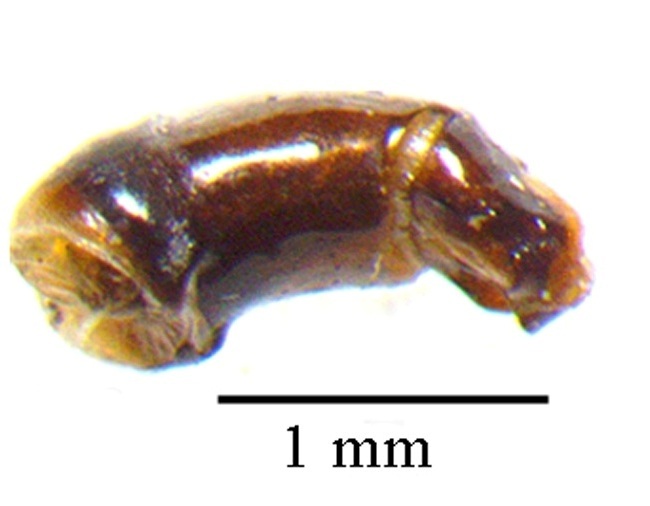


**Figure 3b.**
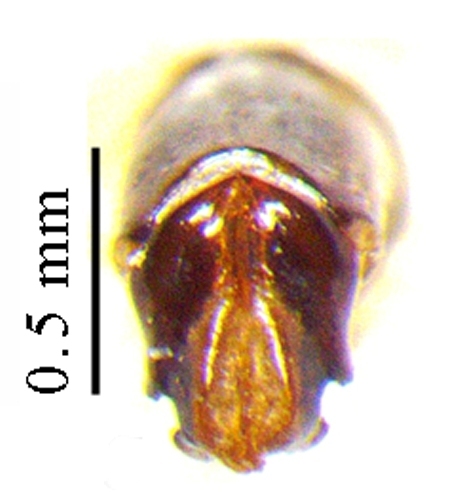


**Figure 3c.**
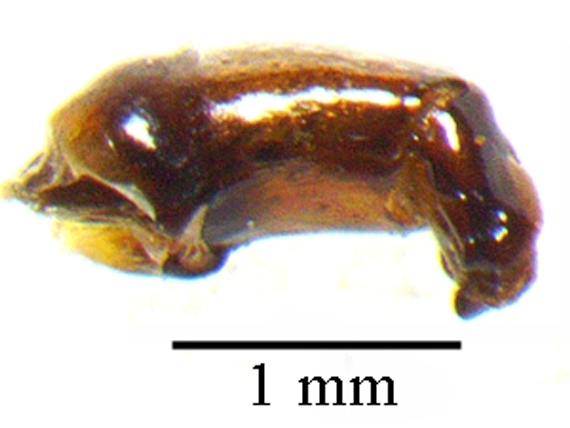


**Figure 3d.**
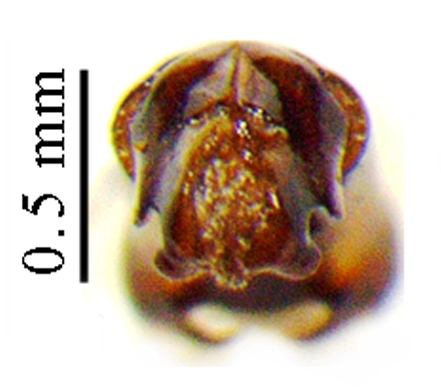


**Table 1.** Preliminary checklist of Indian species of tribe Oniticellini Kolbe, 1905.

Sl. No.	Species	Distribution		References
		India	World	
Subtribe Drepanocerina van Lansberge, 1875 (Genera - 11, Living species - 46)				
1.	*Eodrepanus striatulus* (Paulian, 1945)	Arunachal Pradesh, Assam, Manipur, Meghalaya	China, Laos, Myanmar, Nepal, Thailand, Vietnam	Biswas (1980),Barbero et al. (2009), Schoolmeesters (2020)
2.	*Sinodrepanus falsus* (Sharp, 1875)	Arunachal Pradesh, Assam, Gujarat, Meghalaya, Rajasthan	China, Laos, Thailand	Sharp (1875), Biswas and Chatterjee (1985), Sewak (2009), Schoolmeesters (2020)
3.	*Tibiodrepanus hircus* (Wiedemann, 1823)	Tamil Nadu	China, Indonesia, Myanmar, Philippines, Sri Lanka, Vietnam	Barbero et al. (2011), Schoolmeesters (2020)
4.	*Tibiodrepanus kazirangensis* (Biswas, 1980)	Assam	No Record found	Biswas (1980), Schoolmeesters (2020)
5.	*Tibiodrepanus setosus* (Wiedemann, 1823)	Chhattisgarh, Haryana, Himachal Pradesh, Karnataka, Kerala, Maharashtra, Madhya Pradesh, Puducherry, Sikkim, Tamil Nadu, Uttarakhand, Uttar Pradesh, West Bengal	Bhutan, China, Indonesia, Laos, Myanmar, Nepal, Pakistan, Philippines, Sri Lanka, Thailand, Vietnam	Löbl and Smetana (2006), Barbero et al. (2011), Thomas et al. (2011), Gajendra and Prasad (2016), Singh et al. (2017), Schoolmeesters (2020)
6.	*Tibiodrepanus sinicus* (Harold, 1868)	Bihar, Gujarat, Himachal Pradesh, Madhya Pradesh, Tamil Nadu, Uttarakhand	Bhutan, China, Indonesia, Laos, Malaysia, Myanmar, Nepal, Pakistan, Philippines, Sri Lanka, Thailand, Vietnam	Löbl and Smetana (2006), Barbero et al. (2011), Thomas et al. (2011), Sathiandran et al. (2015), Schoolmeesters (2020)
Subtribe Oniticellina Kolbe 1905; Genera- 10, Living species- 91				
7.	*Euoniticellus pallens* (Olivier, 1789)	Gujarat, Haryana, Himachal Pradesh, Punjab, Rajastan, Uttarakhand, Uttar Pradesh	Afghanistan, Algeria, Armenia, Egypt, Ethiopia, France, Georgia, Iran, Iraq, Italy, Kazakhstan, Kuwait, Libya, Morocco, Oman, Pakistan, Senegal, Somalia, Spain, Tajikistan, Turkey, Turkmenistan, Uzbekistan, Yemen	Löbl and Smetana (2006), Chandra et al. (2012), Schoolmeesters (2020), Siddiqui et al. (2014)
8.	*Euoniticellus pallipes* (Fabricius, 1781)	Gujarat, Haryana, Himachal Pradesh, Jammu & Kashmir, Maharashtra, Rajasthan, Tamil Nadu, Uttarakhand, Uttar Pradesh, West Bengal	Afghanistan, Albania, Algeria, Armenia, Australia, Austria, Bangladesh, Bhutan, Bosnia, Bulgaria, China, Cyprus, Egypt, Ethiopia, France, Georgia, Greece, Hungary, Iran, Iraq, Israel, Italy, Kazakhstan, Kuwait, Libya, Mongolia, Morocco, Oman, Pakistan, Russia, Senegal, Somalia, Spain, Tajikistan, Turkey, Turkmenistan, Uzbekistan	Löbl and Smetana (2006), Thomas et al. (2011), Chandra et al. (2012), Sullivan et al. (2016), Singh et al. (2017), Schoolmeesters (2020)
9.	*Liatongus affinis* (Arrow, 1908)	Assam, Karnataka, Manipur, Tripura, West Bengal	China, Myanmar, Thailand	Löbl and Smetana (2006), Arrow (1931), Chatterjee and Biswas (2000), Karimbumkara and Priyadarsanan (2013), Schoolmeesters (2020)
10.	*Liatongus gagatinus* (Hope, 1831)	Arunachal Pradesh, Assam, Himachal Pradesh, Kashmir, Meghalaya, Nagaland, Sikkim, Uttarakhand, West Bengal	China, Laos, Myanmar, Nepal, Sudan, Thailand, Vietnam	Löbl and Smetana (2006), Arrow (1931), Biswas and Chatterjee (1985), Chandra (2005), Sewak (2006), Chandra and Gupta (2012), Chandra et al. (2012), Kabakov and Shokhin (2014),Schoolmeesters (2020)
11.	*Liatongus indicus* (Arrow, 1908)	Karnataka, Kerala, Tamil Nadu	No Record Found	Arrow (1931), Thomas et al. (2011), Anto and Vinod (2017), Schoolmeesters (2020)
12.	*Liatongus martialis* (Harold, 1879)	Gujarat, Rajastan, Uttar Pradesh	Myanmar	Arrow (1931), Sewak (2009), Schoolmeesters (2020)
13.	*Liatongus mergacerus* (Hope, 1831)	Arunachal Pradesh, Sikkim, Uttarakhand, Uttar Pradesh, West Bengal	Nepal, Bhutan, Sudan	Löbl and Smetana (2006), Arrow (1931), Biswas and Chatterjee (1985), Sewak (2006), Schoolmeesters (2020)
14.	*Liatongus minutus* (Motschulsky, 1860)	Arunachal Pradesh	China, Japan, North Korea, Russia, South Korea	Biswas and Chatterjee (1985), Bezborodov (2007), Kabakov and Shokhin (2014), Schoolmeesters (2020)
15.	*Liatongus phanaeoides* (Westwood, 1839)	Arunachal Pradesh, Himachal Pradesh, Punjab, Uttarakhand, West Bengal	China, Japan, Myanmar, North Korea, Pakistan, South Korea, Thailand	Arrow (1931), Biswas and Chatterjee (1985), Chandra (2005), Sewak (2006), Chandra and Gupta (2012), Chandra et al. (2012), Schoolmeesters (2020)
16.	*Liatongus rhinoceros* (Arrow, 1931)	Himachal Pradesh, Punjab, Sikkim, West Bengal	Nepal, Sudan	Löbl and Smetana (2006), Arrow (1931), Chandra (2005), Schoolmeesters (2020)
17.	*Liatongus triacanthus* (Boucomont, 1920)	Sikkim, West Bengal	China, Myanmar, Sudan	Löbl and Smetana (2006), Schoolmeesters (2020)
18.	*Liatongus vertagus* (Fabricius, 1798)	Arunachal Pradesh, Assam, Himachal Pradesh, Manipur, Uttar Pradesh	China, Myanmar, Thailand	Chandra (2005), Löbl and Smetana (2006), Sewak (2006), Chandra and Gupta (2012), Chandra et al. (2012), Schoolmeesters (2020)
19.	*Oniticellus cinctus* ((Fabricius, 1775)	Arunachal Pradesh, Assam, Bihar, Chhattisgarh, Gujarat, Haryana, Himachal Pradesh, Karnataka, Kashmir, Kerala, Madhya Pradesh, Maharashtra, Tamil Nadu, Tripura, Uttarakhand, Uttar Pradesh, West Bengal	Bangladesh, China, Indonesia, Malaysia, Myanmar, Pakistan, Thailand, Vietnam	Arrow (1931), Biswas and Chatterjee (1985), Chandra (2005), Löbl and Smetana (2006), Sewak (2006), Chandra and Ahirwar (2007), Sewak (2009), Thomas et al. (2011), Karimbumkara and Priyadarsanan (2013), Kabakov and Shokhin (2014), Anto and Vinod (2017), Singh et al. (2017), Schoolmeesters (2020)
20.	*Oniticellus gayeni* Biswas & Chatterjee, 1985	Arunachal Pradesh	No Record Found	Biswas and Chatterjee (1985), Löbl and Smetana (2006), Sewak (2006), Schoolmeesters (2020)
21.	*Oniticellus namdaphaensis* Biswas & Chatterjee, 1985	Arunachal Pradesh	No Record Found	Biswas and Chatterjee (1985), Löbl and Smetana (2006), Sewak (2006), Schoolmeesters (2020)
22.	*Oniticellus subhendui* Biswas & Chatterjee, 1985	Arunachal Pradesh	No Record Found	Biswas and Chatterjee (1985), Löbl and Smetana (2006), Sewak (2006), Schoolmeesters (2020)
23.	*Tiniocellus spinipes* (Roth, 1851)	Chhattisgarh, Gujarat, Haryana, Himachal Pradesh, Karnataka, Kerala, Madhya Pradesh, Maharashtra, Uttarakhand, Uttar Pradesh	Angola, Botswana, Burkina Faso, Eritrea, Ethiopia, Ghana, Ivory Coast, Kenya, Malawi, Mozambique, Namibia, Pakistan, Republic Democratic Congo, Republic of Guinea, Republic South Africa, Senegal, Somalia, Tanzania, Uganda, Zambia, Zimbabwe	Péringuey (1901), Löbl and Smetana (2006), Branco (2010), Thomas et al. (2011), Chandra and Gupta (2013), Karimbumkara and Priyadarsanan (2013), Noureen et al. (2015), Gajendra and Prasad (2016), Philips (2016), Anto and Vinod (2017), Singh et al. (2017), Schoolmeesters (2020)
24.	*Tiniocellus imbellis* (Bates, 1891)	Bihar, Himachal Pradesh, Karnataka, Kerala, Madhya Pradesh, Maharashtra, Punjab, Sikkim, Tamil Nadu, Uttar Pradesh, West Bengal	Nepal, Pakistan	Löbl and Smetana (2006), Branco (2010), Schoolmeesters (2020), Siddiqui et al. (2014)
